# Structure of the *Saccharolobus solfataricus* GINS tetramer

**DOI:** 10.1107/S2053230X25003085

**Published:** 2025-04-16

**Authors:** Srihari Shankar, Eric J. Enemark

**Affiliations:** a Department of Biochemistry and Molecular Biology University of Arkansas for Medical Sciences 4301 West Markham Street, Slot 516 Little Rock AR72205 USA; b Winthrop P. Rockefeller Cancer Institute University of Arkansas for Medical Sciences Little Rock AR72205 USA; University of Toronto, Canada

**Keywords:** DNA replication, GINS, minichromosome maintenance, archaea

## Abstract

DNA replication is tightly regulated to ensure genomic stability and prevent several diseases, including cancers. Eukaryotes and archaea partly achieve this regulation by strictly controlling the activation of hexameric minichromosome maintenance (MCM) helicase rings that unwind DNA during its replication. In eukaryotes, MCM activation critically relies on the sequential recruitment of the essential factors Cdc45 and a tetrameric GINS complex at the onset of the S-phase to generate a larger CMG complex. We present the crystal structure of the tetrameric GINS complex from the archaeal organism *Saccharolobus solfataricus* (*Sso*) to reveal a core structure that is highly similar to the previously determined GINS core structures of other eukaryotes and archaea. Using molecular modeling, we illustrate that a subdomain of *Sso*GINS would need to move to accommodate known interactions of the archaeal GINS complex and to generate a *Sso*CMG complex analogous to that of eukaryotes.

## Abstract 2

Archaeal organisms possess Cdc45 and GINS homologs that are likely to serve similar functional roles, but their structural interactions with the MCM helicase and hence their mechanism of MCM activation are not as well understood as for their eukaryotic counterparts. We present the crystal structure of the *S. solfataricus* GINS tetrameric complex and illustrate that a subdomain would need to move to accommodate known archaeal GINS-complex interactions and to generate an *S. solfataricus* CMG complex analogous to that of eukaryotes.

## 1. Introduction

DNA replication is the fundamental life process of all organisms where genetic material is duplicated in preparation for cell division. This process is tightly regulated to ensure that DNA is fully and faithfully replicated only one time each cell division (Bell & Labib, 2016 ▸; Stillman, 2005 ▸). Disruptions that over-replicate, under-replicate or inaccurately replicate DNA lead to several serious diseases, including cancers (Bell & Labib, 2016 ▸; Stillman, 2005 ▸). One way that cells maintain this strict control is via the cell cycle, which temporally determines when cells begin to synthesize new DNA and when it safe to segregate the copied DNA to daughter cells (Bell & Labib, 2016 ▸; Stillman, 2005 ▸).

The molecular basis for the initiation of DNA replication involves local melting of DNA where some base pairs are opened to allow the establishment of an active helicase–DNA species that becomes competent to unwind and separate the two strands of the DNA double helix (Bell & Labib, 2016 ▸). The separated strands are used by DNA polymerases in the synthesis of new DNA. Bacteria and some viruses separate the two strands and then load a six-membered ring helicase to encircle the exposed single-stranded DNA (ssDNA) to initiate DNA unwinding (Kaguni, 2011 ▸; Bell & Kaguni, 2013 ▸). In contrast, eukaryotes and archaea load a six-membered ring helicase, the minichromosome maintenance (MCM) complex, to encircle DNA and subsequently activate this MCM–DNA species to become competent for unwinding DNA (Bell & Labib, 2016 ▸). In the case of yeast (*Saccharomyces cerevisiae*, *Sc*), and likely all eukaryotes, this activation critically relies on the sequential recruitment of Cdc45 and the tetrameric GINS complex (Moyer *et al.*, 2006 ▸). Cdc45 and GINS factors interact directly with the MCM complex and with each other to form a larger CMG complex (Cdc45–MCM–GINS; Yuan *et al.*, 2016 ▸). Following the recruitment of both Cdc45 and GINS, initial DNA unwinding is observed biochemically (Douglas *et al.*, 2018 ▸) and structurally (Lewis *et al.*, 2022 ▸).

Archaea also use a six-membered MCM ring to unwind DNA (Kelman *et al.*, 2020 ▸), and archaea possess GINS and Cdc45 homologs (Marinsek *et al.*, 2006 ▸; Makarova *et al.*, 2012 ▸) that stimulate the activities of MCM in biochemical DNA-unwinding assays (Yoshimochi *et al.*, 2008 ▸; Xu *et al.*, 2016 ▸; Lang & Huang, 2015 ▸). Crystal structures of the archaeal proteins confirmed that archaeal RecJ (Oyama *et al.*, 2016 ▸; Li *et al.*, 2017 ▸; Oki *et al.*, 2022 ▸) and archaeal GINS (Oyama *et al.*, 2011 ▸) are indeed structural homologs of eukaryotic Cdc45 (Simon *et al.*, 2016 ▸) and GINS (Choi *et al.*, 2007 ▸; Kamada *et al.*, 2007 ▸; Chang *et al.*, 2007 ▸), respectively, suggesting fundamentally conserved mechanisms of action. Several structures have been determined of eukaryotic CMG from multiple organisms and in multiple configurations to illuminate a wealth of mechanistic detail (Yuan *et al.*, 2016 ▸; Georgescu *et al.*, 2017 ▸; Eickhoff *et al.*, 2019 ▸; Rzechorzek *et al.*, 2020 ▸; Baretić *et al.*, 2020 ▸; Yuan *et al.*, 2020 ▸; Lewis *et al.*, 2022 ▸; Xia *et al.*, 2023 ▸; Jenkyn-Bedford *et al.*, 2021 ▸; Cvetkovic *et al.*, 2023 ▸; Henrikus *et al.*, 2024 ▸). Structures from archaeal organisms have been limited, and no structural details of potential interactions between archaeal MCM and either GINS or RecJ have been elucidated. As a stepping stone towards elucidating a potential archaeal CMG complex, we have determined the crystal structure of the tetrameric GINS complex from the archaeal organism *Saccharolobus solfataricus* (*Sso*), an organism for which we have determined multiple MCM structures in several states using X-ray crystallography (Miller *et al.*, 2014 ▸; Meagher *et al.*, 2019 ▸) and cryo-EM (Meagher *et al.*, 2022 ▸). The core of the *Sso*GINS structure is highly conserved with other GINS structures determined from eukaryotic and archaeal organisms. One subdomain adopts an apparently stable conformation that would need to rearrange to permit the assembly of a CMG complex analogous to that of eukaryotes.

## 2. Materials and methods

### 2.1. Macromolecule production

The two genes encoding the GINS proteins of *S. solfataricus* were amplified from genomic DNA (ATCC 35092D-5) by polymerase chain reaction (PCR) and cloned into the two expression sites of pETDuet (Novagen). The gene encoding *Sso*GINS23 (NCBI AZF71192.1) was cloned between the BamHI and SalI sites of multiple cloning site 1, which provided an N-terminal hexahistidine tag. The gene encoding *Sso*GINS51 (AAK41311.1) was cloned between the NdeI and XhoI sites of multiple cloning site 2. The hexahistidine tag was genetically deleted from the construct by PCR amplification of the plasmid (see Table 1 ▸), including one phosphorylated primer, followed by circular ligation and transformation to yield the expression plasmid used in this study (pEE093).

Plasmid pEE093 was freshly transformed into *Escherichia coli* BL21(DE3)-RIPL cells and seeded into a 100 ml overnight culture in LB medium supplemented with 2% glucose and 50 mg l^−1^ ampicillin. Following overnight growth, the 100 ml culture was distributed into 6 l LB medium supplemented with 0.4% glucose and 50 mg l^−1^ ampicillin. The cultures were grown in a shaker/incubator at 37°C until the OD reached 0.4, and the temperature was reduced to 18°C. When the OD of the culture had reached 0.6–0.9, isopropyl β-d-1-thiogalactopyranoside (IPTG) was added to a 0.4 m*M* concentration to induce expression of the two proteins. After 18 h at 18°C, the cells were harvested by centrifugation and were resuspended in buffer 1 (50 m*M* Tris pH 8.3, 250 m*M* NaCl, 10% glycerol, Roche EDTA-free protease inhibitor). The cells were lysed on ice with one pass through a microfluidizer at 62 MPa. The cellular lysate was heated in a water bath for 15 min at 80°C and the soluble fraction was isolated by centrifugation. The sodium chloride concentration was increased to 1 *M* and 10% polyethylenimine–HCl was added to a 0.3% final concentration to precipitate nucleic acids. The soluble fraction was isolated by centrifugation and subjected to ammonium sulfate precipitation at 4°C by adding 43.6 g ammonium sulfate per 100 ml of solution. Precipitated proteins were isolated by centrifugation, resuspended in buffer 1 and purified by size-exclusion chromatography on an S200 column. The NaCl concentration was decreased by dilution with buffer 2 (20 m*M* Tris pH 8.3, 50 m*M* NaCl, 5% glycerol, 2 m*M* β-mercaptoethanol) and the solution was applied onto a Mono Q column for anion-exchange chromatography. The sample was eluted with a linear gradient from buffer 2 to buffer 3 (20 m*M* Tris pH 8.3, 1 *M* NaCl, 5% glycerol, 2 m*M* β-mercaptoethanol). SDS–PAGE of the fractions indicated two co-eluting bands of similar intensity. Pooled fractions (2 ml) were dialyzed against 20 m*M* Tris pH 8.3, 100 m*M* NaCl and concentrated to 11 mg ml^−1^ for crystallization (based on absorbance at 280 nm; tetramer ɛ_280_ = 57 760 *M*^−1^ cm^−1^; tetramer molecular weight of 76 870.34).

### 2.2. Crystallization

**Figure 1:**
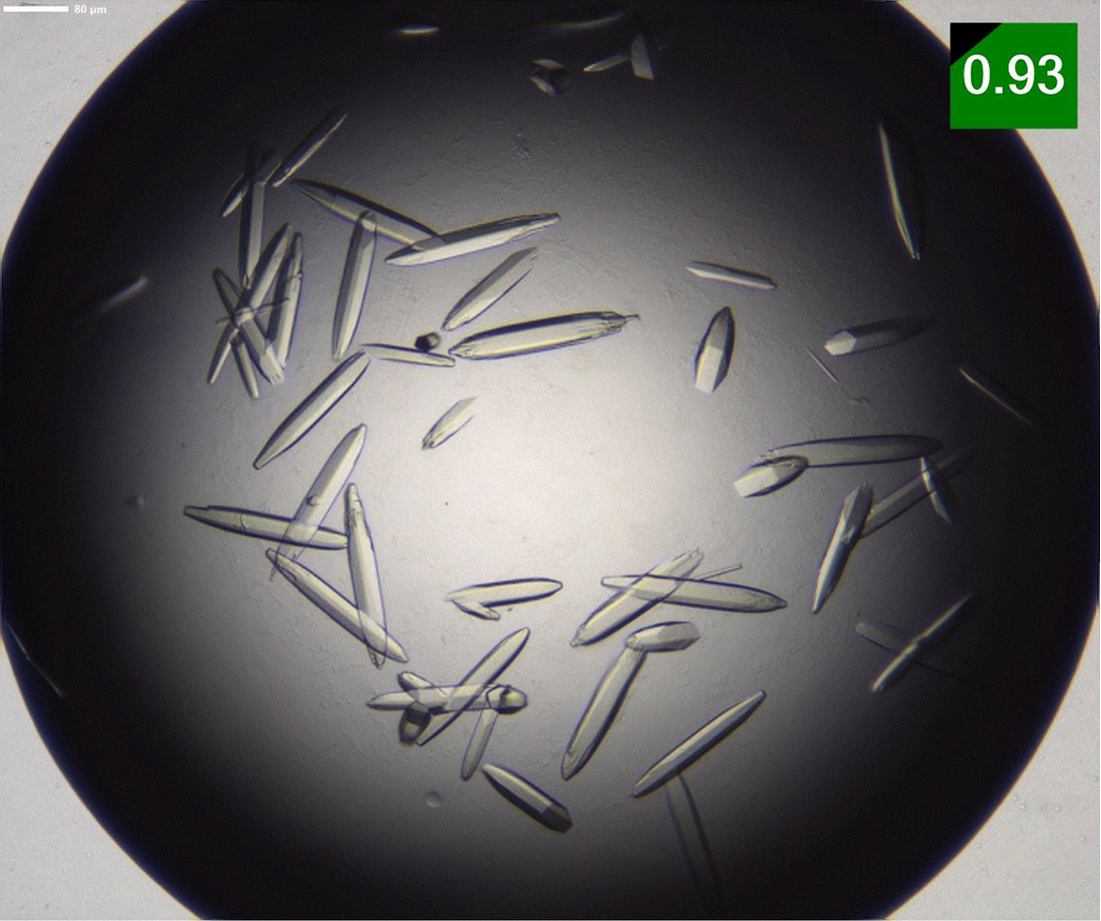
Crystals of *Sso*GINS imaged using a Formulatrix Rock Imager 1000 plate incubator/hotel. The crystallization solution consisted of 0.18 *M* tri­ammonium citrate, 20%(*w*/*v*) PEG 3350.

Crystallization screening was performed with an SPT Labtech Mosquito LCP drop-setting robot using the hanging-drop method with 200 nl protein solution mixed with 200 nl well solution. Crystals grew in 96-well plates over a few days using multiple commercial screens in a Formulatrix plate incubator/hotel at 18°C. Crystals from JCSG solution G1 [0.18 *M* triammonium citrate, 20%(*w*/*v*) PEG 3350] provided suitably large crystals for harvesting and subsequent collection of diffraction data (Fig. 1 ▸, Table 2 ▸).

### 2.3. Data collection and processing

Single crystals were harvested from crystallization drops, quickly passed through 1:3 ethylene glycol:well solution and flash-cooled in liquid nitrogen. Cooled crystals were shipped to SER-CAT beamline 22-ID at the Advanced Photon Source (APS) in a dry shipper for evaluation and data collection. Data were collected from two positions of one crystal using an EIGER 16M detector. Each collection consisted of 360° of total oscillation in 0.25° oscillation widths and 0.1 s exposures with 13.531% beamline transmission. The two data sets were processed, scaled and merged using *HKL*-2000 (Otwinowski & Minor, 1997 ▸) in primitive hexagonal point group 6 with a high-resolution limit of 2.3 Å and 37-fold overall average redundancy (Table 3 ▸). The systematic absence of the odd 00*l* reflections strongly suggested space group *P*6_3_.

### 2.4. Structure solution and refinement

The structure was solved by molecular replacement with *Phaser* (McCoy *et al.*, 2007 ▸), which placed two copies of a monomer of *Sso*GINS23 and two copies of a monomer of *Sso*GINS51 in space group *P*6_3_ (Table 4 ▸). The monomeric search models used for molecular replacement were taken from an *AlphaFold-Multimer* (Evans *et al.*, 2021 ▸) prediction of an (*Sso*GINS23)_2_(*Sso*GINS51)_2_ tetramer. The structure was iteratively refined in *Phenix* (Liebschner *et al.*, 2019 ▸; Afonine *et al.*, 2012 ▸) and rebuilt in *Coot* (Casañal *et al.*, 2020 ▸; Emsley *et al.*, 2010 ▸; Emsley & Cowtan, 2004 ▸). The final refinement included 24 autogenerated TLS groups (four chain *A*, six chain *B*, eight chain *C* and six chain *D*) and secondary-structure restraints. The final structure has an r.m.s.d. of 2.193 Å (2844 matched atoms) from the *AlphaFold-Multimer* (Evans *et al.*, 2021 ▸) model that provided the molecular-replacement search models as calculated in *PyMOL* (Schrödinger).

## 3. Results and discussion

### 3.1. Overall architecture

**Figure 2:**
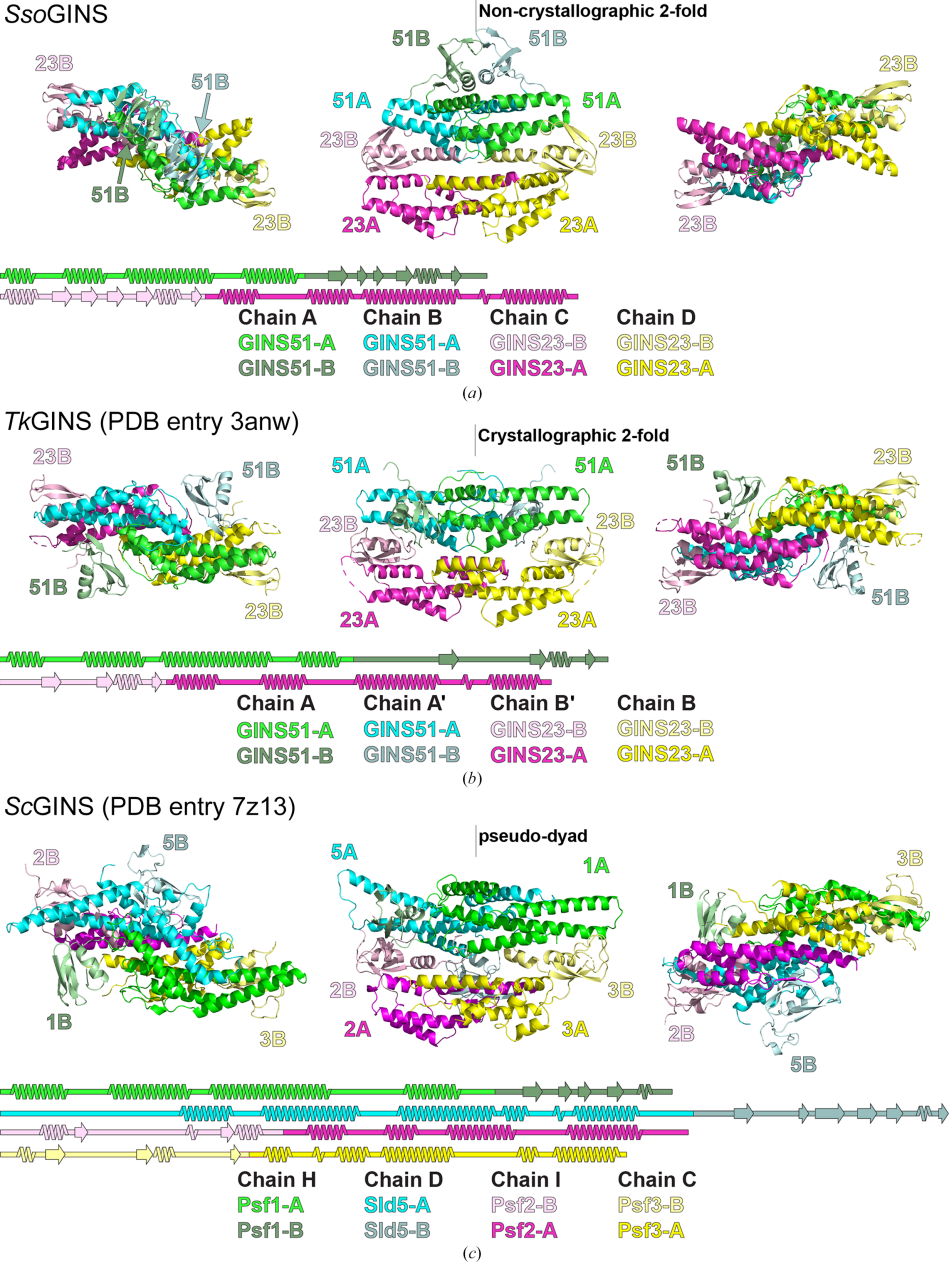
(*a*) The structure of the *Sso*GINS tetramer is an approximately twofold-symmetric assembly of two GINS51 subunits (green and cyan) and two GINS23 subunits (magenta and yellow). Both subunit types consist of two subdomains; one is fully α-helical (A) and the other contains β-strands (B). These subdomains occur in reverse order for the two subunit types. The secondary, tertiary and quaternary structures are highly similar to those previously observed for the GINS complexes from the archaeon *Thermococcus kodakarensis* (*b*) (PDB entry 3anw; Oyama *et al.*, 2011 ▸) and the eukaryotic yeast (*c*) (PDB entry 7z13; Lewis *et al.*, 2022 ▸). The structurally conserved core consists of the full GINS23 subunits (yeast Psf2 and Psf3) and the A subdomains of GINS51 (yeast Sld5 and Psf1). The B subdomains of GINS51 (yeast Sld5 and Psf1) occupy variable positions, indicating that this subdomain may be mobile. Structure images were prepared with *PyMOL* (Schrödinger) and secondary-structure diagrams were prepared with the Google Colab implementation of *SSDraw* (Chen & Porter, 2023 ▸). The *Sso*GINS51A subdomains uniquely form a significant dimeric interaction.

The structure adopts a tetrameric architecture, with two copies of GINS51 and two copies of GINS23 in an approximately twofold-symmetric complex. The structure is highly consistent with other GINS structures (Fig. 2 ▸) despite relatively low sequence identity. *Sso*GINS51 has 21.7% sequence identity to *Thermococcus kodakarensis* (*Tk*) GINS51, 16% identity to *Sc*Sld5 and 15.6% identity to *Sc*Psf1. *Sso*GINS23 has 21.2% sequence identity to *Tk*GINS23, 21.1% identity to *Sc*Psf2 and 18.8% identity to *Sc*Psf3. Each subunit has two subdomains, with a larger α-helical subdomain (A) and a smaller subdomain that contains β-strands (B), consistent with the structures of other archaeal and eukaryotic GINS protein structures (Oyama *et al.*, 2011 ▸; Choi *et al.*, 2007 ▸; Kamada *et al.*, 2007 ▸; Chang *et al.*, 2007 ▸; Yuan *et al.*, 2016 ▸). The order of the A and B subdomains is reversed between the two subunits, with the A subdomain first in *Sso*GINS51 and the B subdomain first in *Sso*GINS23 (Fig. 2 ▸). These subdomain orders are consistent with those of other eukaryotic and archaeal GINS structures (Oyama *et al.*, 2011 ▸; Choi *et al.*, 2007 ▸; Kamada *et al.*, 2007 ▸; Chang *et al.*, 2007 ▸; Yuan *et al.*, 2016 ▸) and with the A/B topologies predicted for the *Sso*GINS subunits (Xu *et al.*, 2016 ▸).

The core tetrameric quaternary structure of the GINS complexes of eukaryotes and archaea (Fig. 2 ▸) is highly conserved. In the archaeal structures, the core consists of both complete GINS23 subunits and the two GINS51A sub­domains. In eukaryotic GINS, the core consists of the complete Psf2 and Psf3 subunits and the helical A subdomains of Sld5 and Psf1. The underlying subdomain structure of GINS51B, Sld5B and Psf1B is conserved, but its placement relative to the GINS tetramer core varies among the structures. In the case of *Sso*GINS, the two GINS51B subdomains have a unique and sizable dimeric interaction surface (at the top of the complex in the central panel of Fig. 2 ▸). This placement was also predicted by *AlphaFold-Multimer* (Evans *et al.*, 2021 ▸), as illustrated in the discussion below.

### 3.2. The *Sso*GINS51B dimeric interface is incompatible with simultaneous polymerase interaction

**Figure 3:**
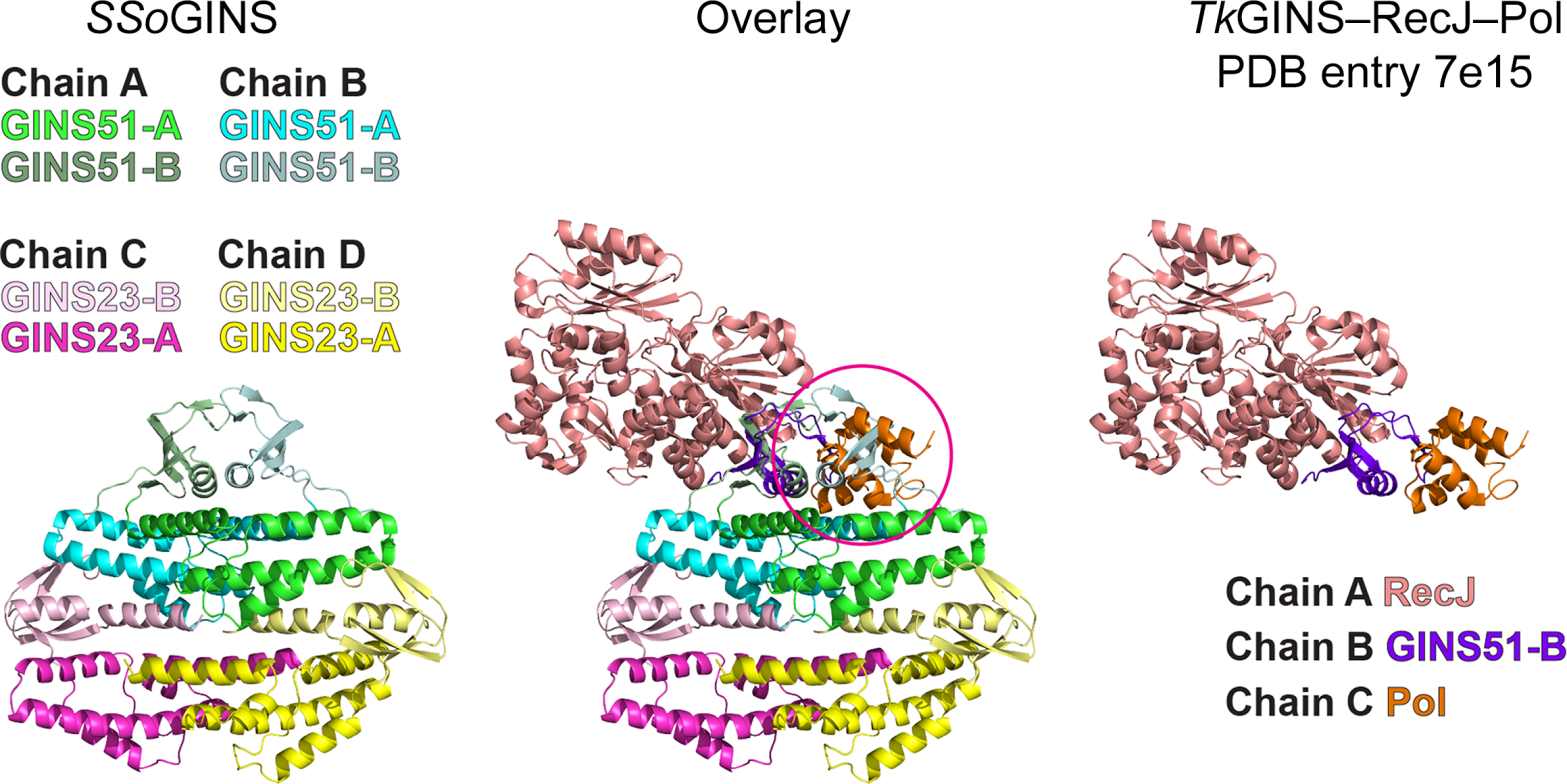
The dimeric interaction of the *Sso*GINS51A subdomains is incompatible with formation of the interaction with polymerase illustrated for *Tk*GINS51B–RecJ–Pol. The GINS51B subunit (purple) of the *Tk*GINS51B–RecJ–Pol structure (PDB entry 7e15; Oki *et al.*, 2022 ▸) was superimposed on one *Sso*GINS51B subdomain of the *Sso*GINS crystal structure to investigate the compatibility of similar interactions. Following this superposition, the RecJ subunit (salmon) did not clash with any component of *Sso*GINS, but the DNA polymerase II small subunit severely clashed with the dyad-related *Sso*GINS51B subunit. Hence, the *Sso*GINS51B dimeric interface is not compatible with simultaneous interaction with DNA polymerase in the fashion of *Tk*GINS–RecJ–Pol. Superpositions and figures were prepared in *PyMOL* (Schrödinger).

The dimeric association of the two *Sso*GINS51B sub­domains was intriguing and was investigated further. In eukaryotes and archaea, the corresponding subdomain interacts structurally with the key replication factors Cdc45/RecJ and with DNA polymerase. In the archaeon *T. kodakarensis*, the GINS51B subdomain interacts with RecJ and with the DNA polymerase II small subunit (PDB entry 7e15; Oki *et al.*, 2022 ▸). In the eukaryotic CMG structure, the GINS Psf1B subdomain similarly interacts with Cdc45 and with DNA polymerase epsilon subunit B (PDB entry 7z13; Lewis *et al.*, 2022 ▸). The crystal structure of *Tk*GINS51–RecJ–Pol was superimposed on one *Sso*GINS51B subdomain to investigate the compatibility of similar interactions within the *Sso*GINS tetramer (Fig. 3 ▸). This superposition suggested that a RecJ subunit could similarly interact with *Sso*GINS51B without clashes, but the polymerase subunit would fully clash with the dyad-related *Sso*GINS51B subdomain (magenta circle in Fig. 3 ▸). We therefore conclude that the dimeric interface of the *Sso*GINS51B subdomains of the crystal structure is not compatible with the simultaneous adoption of an interaction with polymerase as identified for *Tk*GINS.

### 3.3. *AlphaFold* structure prediction of *Sso*GINS–RecJ

**Figure 4:**
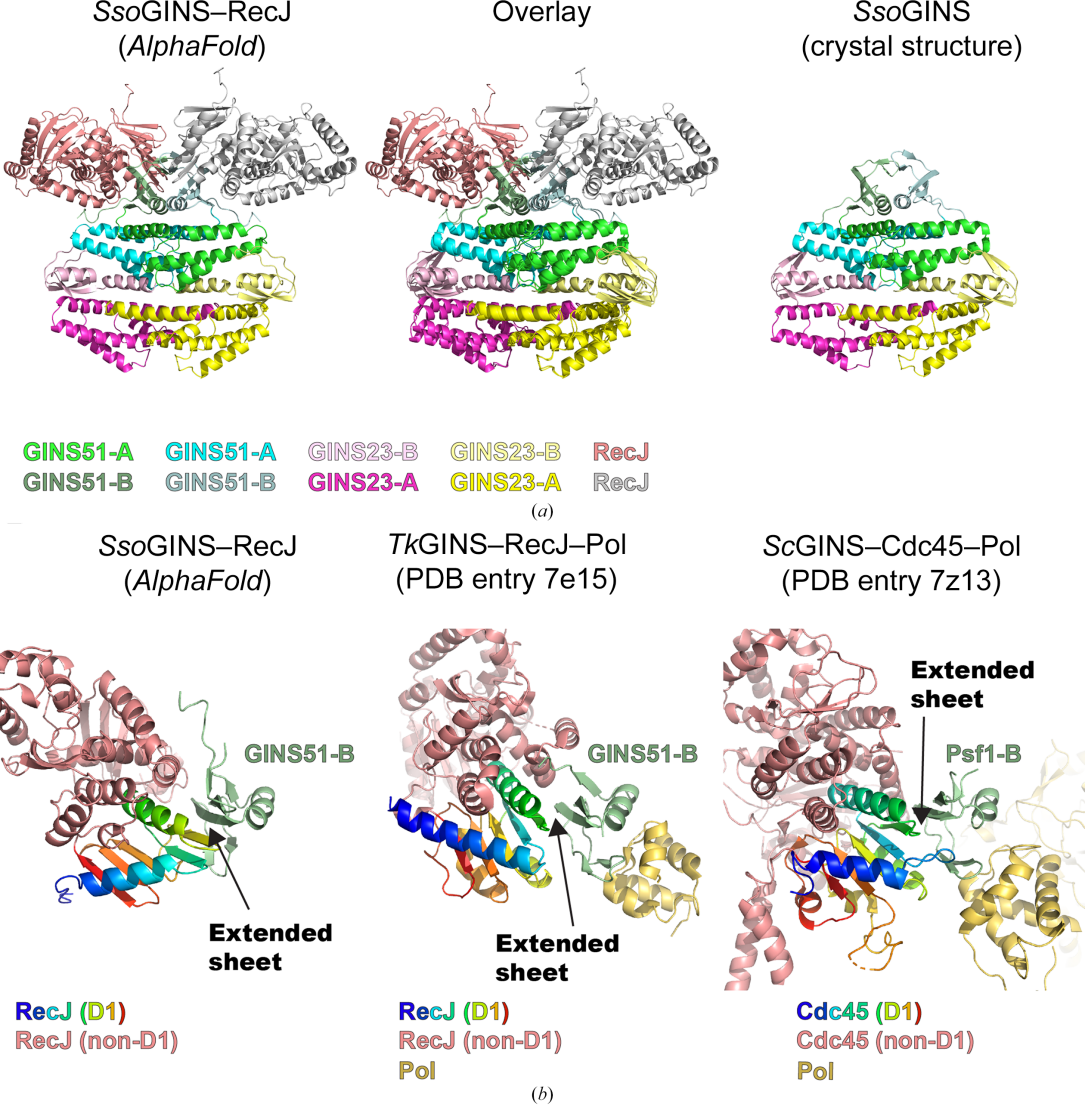
The predicted structure of *Sso*GINS–RecJ shows interaction between *Sso*GINS51B similar to the interactions observed in *Tk*GINS–RecJ and *Sc*GINS. (*a*) The *AlphaFold-Multimer* (Evans *et al.*, 2021 ▸) prediction of the *Sso*GINS_2_RecJ_2_ structure shows an interaction between RecJ and *Sso*GINS51B in a complex that is similar to the superposition in Fig. 3 ▸. (*b*) The mode of interaction is similar to that of archaeal and eukaryotic structures. For each case, the two-stranded antiparallel sheet of the B subdomain (light green) forms an extended sheet with the first domain of Cdc45/RecJ (rainbow). Superpositions and figures were prepared in *PyMOL* (Schrödinger).

The superposition of the structure of *Tk*GINS51–RecJ–Pol onto the crystal structure of *Sso*GINS suggested that the *Sso*GINS structure, including the GINS51B subdomain positions, is compatible with placing *Sso*RecJ analogous to the structure of *Tk*GINS51–RecJ–Pol (Oki *et al.*, 2022 ▸). In yeast, GINS and Cdc45 interact with each other following their sequential recruitment (Douglas *et al.*, 2018 ▸) to the MCM ring to form a CMG complex (Moyer *et al.*, 2006 ▸; Yuan *et al.*, 2016 ▸). To explore the *Sso*GINS–RecJ interaction that potentially exists within an assembled CMG complex, the *Sso*GINS–RecJ structure was predicted with *AlphaFold-Multimer* (Evans *et al.*, 2021 ▸). The prediction used two subunits of *Sso*GINS51, two subunits of *Sso*GINS23 and two subunits of *Sso*RecJ (NCBI WP_009990587.1). It used a database cutoff of 20 January 2023. The predicted structure (Fig. 4 ▸) was high confidence in the underlying structures and in their relative placements (ptm 0.831219297780319; iptm 0.81859624; rank 0.8211208546427338) and had strong positional correlation between the GINS complex and RecJ based on the predicted alignment error plot. The GINS portion of the structure strongly resembles the present crystal structure (compare with Fig. 2 ▸). In the predicted structure, the RecJ subunits interact with *Sso*GINS51B, occupying positions that are very similar to those observed when *Tk*GINS51–RecJ–Pol was superimposed on the *Sso*GINS51B subdomain of the crystal structure (Fig. 3 ▸). The interaction between *Sso*GINS51A and *Sso*RecJ in the predicted structure is very similar to the interactions in other archaeal and eukaryotic structures. All involve the addition of strands of the GINS B subdomain to extend the β-sheet of the first domain of Cdc45/RecJ. Hence, the *Sso*GINS–RecJ interaction of the *AlphaFold-Multimer* model is consistent with the interactions observed in both archaea and eukaryotes and would not sterically clash with the conformation of the *Sso*GINS crystal structure.

### 3.4. Modeled changes for compatibility with eukaryotic Mcm2-7–Cdc45–GINS

**Figure 5:**
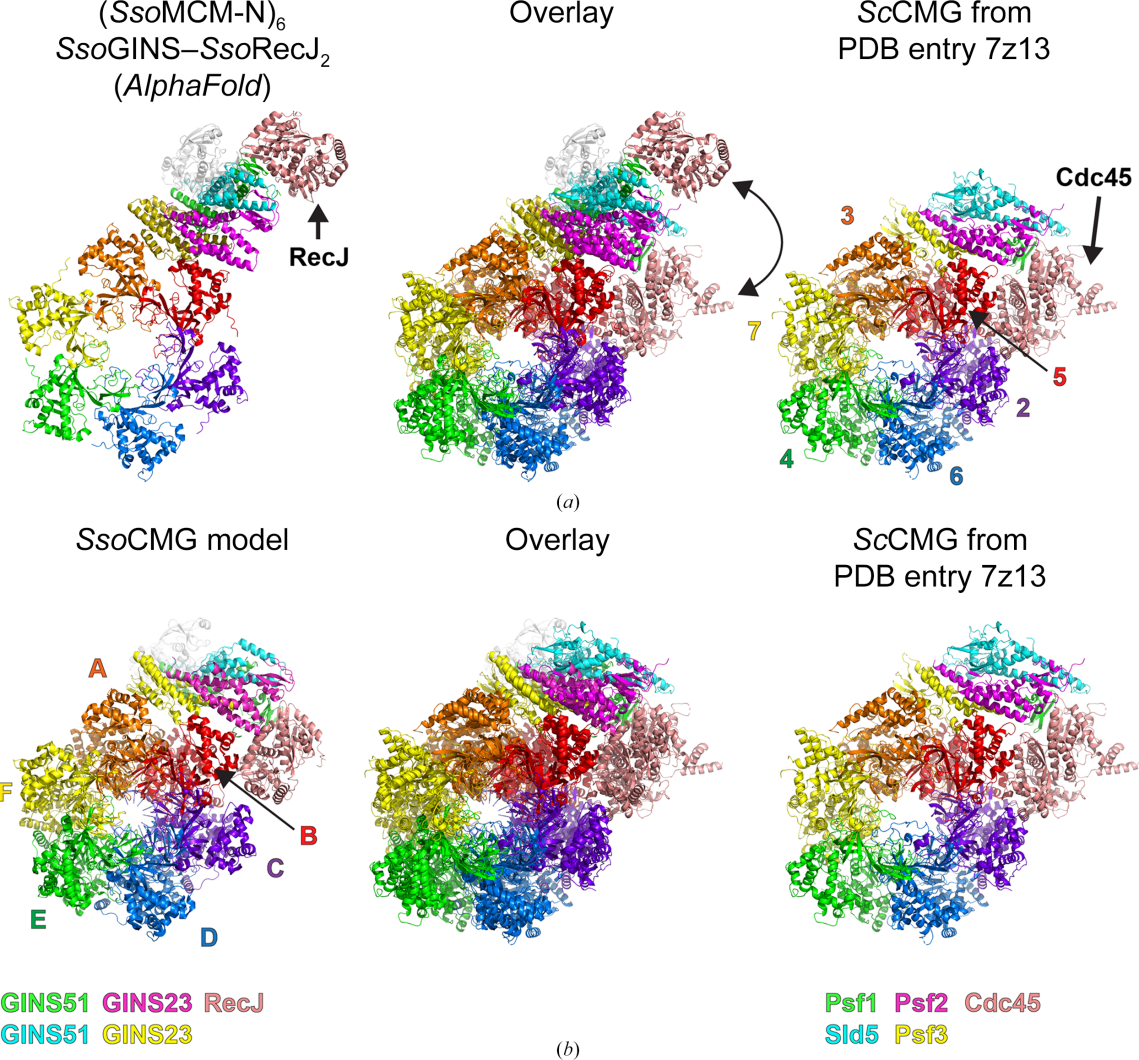
Generation of a model for *Sso*CMG. (*a*) A model for the structure of the *Sso*MCM N-terminal domain hexamer bound to *Sso*GINS and *Sso*RecJ predicted by *AlphaFold-Multimer* (Evans *et al.*, 2021 ▸) is illustrated on the left. The *Sc*CMG portion of PDB entry 7z13 (Lewis *et al.*, 2022 ▸) is illustrated on the right and the overlay of the two is shown in the center. The MCM subunits are colored purple, blue, green, yellow, orange and red. The GINS subunits are colored yellow, magenta, green and cyan in the same scheme as the other figures. RecJ/Cdc45 subunits are in salmon and light gray as in the other figures. The MCM–GINS portions are consistent between the two structures. The *Sso*RecJ subunit interacts with GINS51B as in Fig. 4 ▸(*a*), which places it opposite the MCM ring rather than adjacent as in *Sc*CMG. (*b*) A model for *Sso*CMG was generated from the structures in (*a*) by superimposing one of the *Sso*GINS51B–RecJ units (green and salmon) on the Psf1B and Cdc45 subunits of *Sc*CMG and regularizing the *Sso*GINS51 linker. The ATPase domains of *Sso*MCM were added by superimposing the structure of the nearly full-length *Sso*MCM hexamer crystal structure (PDB entry 6mii; Meagher *et al.*, 2019 ▸). Superpositions and figures were prepared in *PyMOL* (Schrödinger).

To assess the interactions among the components of a potential *Sso*CMG complex, an *AlphaFold-Multimer* prediction was generated using six copies of the *Sso*MCM N-terminal domain, two copies of *Sso*GINS51, two copies of *Sso*GINS23 and two copies of *Sso*RecJ. The prediction used a database cutoff of 20 January 2023. The MCM N-terminal domain was used rather than full-length MCM to save computational time and because Mcm2-7 interacts with Cdc45–GINS exclusively via the Mcm2-7 N-terminal domains in the yeast CMG complex. The predicted model (ptm 0.5662312904705491; iptm 0.5284666; rank 0.5360195239327329) is highly similar to *Sc*CMG in the interactions between MCM and GINS (Fig. 5 ▸*a*). In the predicted model, the *Sso*GINS51B–RecJ positions match those of the *Sso*GINS–RecJ prediction (see Fig. 4 ▸), which places the RecJ subunit at the opposite end of GINS from the MCM ring and not adjacent.

Superpositions with existing structures generated a potential model for *Sso*CMG that included the MCM ATPase domains and placed the RecJ unit adjacent to the MCM ring as in *Sc*CMG. The *Sso*CMG prediction (above) was first aligned with *Sc*CMG (PDB entry 7z13; Lewis *et al.*, 2022 ▸) based on all six OB-folds of the MCM subunits, which generated the superposition in Fig. 5 ▸(*a*). Next, one *Sso*51B–RecJ unit was superimposed on the Psf1–Cdc45 unit of *Sc*CMG. The linker between the 51A and 51B subdomains was regularized in *Coot* (Casañal *et al.*, 2020 ▸; Emsley *et al.*, 2010 ▸; Emsley & Cowtan, 2004 ▸) to illustrate that the linker was sufficiently long to allow the envisioned repositioning of *Sso*51B–RecJ. Lastly, a nearly full-length crystal structure of *Sso*MCM (PDB entry 6mii; Meagher *et al.*, 2019 ▸) was superimposed on the *AlphaFold* model based on all six OB-folds to provide positions of the ATPase domains. The final model (Fig. 5 ▸*b*) represents our best prediction for a potential *Sso*CMG complex analogous to that of eukaryotes. The *AlphaFold*-predicted *Sso*CMG model of Fig. 5 ▸(*a*) was morphed to the eukaryotic-like *Sso*CMG model of Fig. 5 ▸(*b*) with *PyMOL *(Schrödinger) using its *rigimol* routine to help illustrate the molecular difference of the *Sso*51B–RecJ positions (Supplementary Video S1). The 51B subdomain center of mass in the form in Fig. 5 ▸(*a*) differs from that in Fig. 5 ▸(*b*) by 34.4 Å. Center of masses were calculated in *PyMOL* (Schrödinger). Although it is not known whether an *Sso*CMG structure would closely match eukaryotic CMG structures, this analysis shows that the 51B subdomain would need to move a large distance from its crystal structure position to accommodate a eukaryote-like CMG structure.

## 4. Concluding remarks

The large movement described above for the 51B sub­domain is currently speculative because the specific structural form of a potential *Sso*CMG complex is not known. The two predicted structural arrangements strongly differ in the proximity of two 51B subdomains, which provides a basis for a fluorescence-quenching assay similar to those used to study ring opening in DNA sliding clamps (Paschall *et al.*, 2011 ▸; Thompson *et al.*, 2012 ▸). For such an assay, a fluorescent label could be incorporated into the 51B subdomain close to the twofold-symmetric interface of the crystal structure. In the twofold-symmetric form (as in Fig. 5 ▸*a*) of the crystal structure (Fig. 2 ▸), the fluorescent groups of the two 51B subdomains are expected to self-quench due to their close proximity. In a eukaryotic-like CMG form (as in Fig. 5 ▸*b*), measurable fluorescence is anticipated because the two 51B subdomains would be far apart. This loss-of-quenching assay could potentially allow dissection of the mobility of 51B subdomains and the biochemical events that lead to mobility, and is potentially adaptable to a single-molecule format.

## Supplementary Material

PDB reference: *Saccharolobus solfataricus* GINS tetramer, 9moj

Supplementary Movie S1. Remodeling of the SsoGINS51B-RecJ unit (green and salmon) from the AlphaFold-Multimer predicted model of SsoGINS-SsoRecJ (see Fig. 5a) that is needed to generate a SsoCMG model comparable to eukaryotic CMG (see Fig. 5b). The light grey RecJ subunit of Fig. 5 is omitted for clarity. The SsoCMG model was generated by superposition of SsoGINS51B-RecJ on the positions of Psf1-Cdc45 molecules of ScCMG in PDB entry 7z13. DOI: 10.1107/S2053230X25003085/pg5096sup1.mp4

## Figures and Tables

**Table 1:** Macromolecule-production information

Source organism	*Saccharolobus solfataricus*
DNA source	Genomic DNA (ATCC 35092D-5)
Forward-gins51 (NdeI)	gcagggcatatgTTAGATGAATTGGTTAAAAAGGAATTATCAGAGGAAGAAATTACTGAAATTAAGCTAGAGGAAATAATCAAGT
Reverse-gins51 (XhoI)	ccgaggctcgagTCATAATTGTTCTTCAATATCTATCTTATAGGGAGTTAAATAACTTGCTATTATTAATGGCAAGGCTTCTCT
Forward-gins23 (BamHI)	gcagggggatccgATGATCGAAGTAAAACTCAGAGCTATTAAAAGATTGTCAAATGTTTACACTCGCC
Reverse-gins23 (SalI)	ccgagggtcgacCTAGGAATTTCCAATTATATCACCATATAATTCCTTAATAAGTTGTTTTATTGTCTTATAGATTAAAAGCTCTTCTTCTGTCATTCC
pETDuet-MCS1-R	[PHOS]GGTATATCTCCTTCTTAAAGTTAAACAAAATTATTTCTAGAGGGGA
pSSOGINS23-F1	ATGATCGAAGTAAAACTCAGAGCTATTAAAAGATTGTCAAATG
Cloning vector	pETDuet
Expression vector	pETDuet
Expression host	*Escherichia coli* BL21(DE3)-RIPL
Complete amino-acid sequence of the construct produced	
GINS51	MLDELVKKELSEEEITEIKLEEIIKYITLIKKSKTFVSSEIRKEELKFLSELAESLFELRLSKVLEGKVGKGFDEFIFDIFKILKQFYVDLLTGRYIIYNDKIYCIVQKPLIYNDHRVNEGDVLVLPMREALPLIIASYLTPYKIDIEEQL
GINS23	MIEVKLRAIKRLSNVYTRRVMIIEDWNGSSITTGNIELVKGSENQLPQWLAIILEGKKVAKIEDKISIEDLGRILFQERQNMNTPASLVPLGKDFTSRVQLYLETLRKDNNVESLEKLRKSIGILNEIIKIRLRKLIQLAFLNIDDQNLINGMTEEELLIYKTIKQLIKELYGDIIGNS

**Table 2:** Crystallization

Method	Vapor diffusion, hanging drop
Plate type	96-well
Temperature (K)	291
Protein concentration (mg ml^−1^)	11 (based on absorbance at 280 nm)
Buffer composition of protein solution	20 m*M* Tris pH 8.3, 100 m*M* NaCl
Composition of reservoir solution	0.18 *M* triammonium citrate, 20%(*w*/*v*) PEG 3350.
Volume and ratio of drop	200 nl:200 nl
Volume of reservoir (µl)	100

**Table 3:** Data collection and processing Values in parentheses are for the outer shell.

Diffraction source	SER-CAT beamline 22-ID, APS
Wavelength (Å)	1.0
Temperature (K)	100
Detector	EIGER 16M
Crystal-to-detector distance (mm)	300
Rotation range per image (°)	0.25
Total rotation range (°)	360 × 2
Exposure time per image (s)	0.1
Space group	*P*6_3_
*a*, *b*, *c* (Å)	180.24, 180.24, 51.20
α, β, γ (°)	90, 90, 120
Mosaicity (°)	0.107–0.283
Resolution range (Å)	50–2.30 (2.34–2.30)
Total No. of reflections	1581846
No. of unique reflections	42463 (1957)
Completeness (%)	99.2 (91.5)
Multiplicity	37.3 (19.0)
〈*I*/σ(*I*)〉	34.5 (1.0)†
*R*_r.i.m._	0.013 (0.224)
Overall *B* factor from Wilson plot (Å^2^)	52.69

† 〈*I*/σ(*I*)〉 is 1.5 in the 2.38–2.34 Å bin, 1.5 in the 2.43–2.38 Å bin and 4 in 2.48–2.43 Å bin.

**Table 4:** Structure refinement Values in parentheses are for the outer shell.

Resolution range (Å)	31.22–2.30 (2.36–2.30)
Completeness (%)	91.1
σ Cutoff	All data
No. of reflections, working set	37080 (2256)
No. of reflections, test set	1904 (112)
Final *R*_cryst_	0.220 (0.3043)
Final *R*_free_	0.243 (0.3716)
Cruickshank DPI	n.d.
No. of non-H atoms	
Protein	5304
Ligand	0
Water	5
R.m.s. deviations	
Bond lengths (Å)	0.002
Angles (°)	0.386
Average *B* factors (Å^2^)	
Protein	79.6
Ramachandran plot	
Most favored (%)	98.43
Allowed (%)	1.57
